# Supplementary material for: Metagenomic insights into microbial community structure and metabolism in alpine permafrost on the Tibetan Plateau
Source: Nat Commun. 2024 Jul 14;15:5920. doi: 10.1038/s41467-024-50276-2 (PMC11247091; doi:10.1038/s41467-024-50276-2)
Supplement: Supplementary file 1 — Supplementary Information [file 41467_2024_50276_MOESM1_ESM.pdf]

Supplementary Information for

Metagenomic insights into microbial community structure and metabolism in alpine permafrost on the Tibetan Plateau

Luyao Kang[1,2,3], Yutong Song[1,2,3], Rachel Mackelprang[4], Dianye Zhang[1,2], Shuqi Qin[1,2], Leiyi Chen[1,2], Linwei Wu[5], Yunfeng Peng[1,2] and Yuanhe Yang[1,2,3*]

[1]State Key Laboratory of Vegetation and Environmental Change, Institute of Botany, Chinese Academy of Sciences, Beijing 100093, China.
[2]China National Botanical Garden, Beijing 100093, China
[3]University of Chinese Academy of Sciences, Beijing 100049, China.
[4]California State University Northridge, 18111 Nordhoff St., Northridge, CA 91330, USA.
[5]Institute of Ecology, Key Laboratory for Earth Surface Processes of the Ministry of Education, College of Urban and Environmental Sciences, Peking University, Beijing 100871, China.

[*]**Corresponding author:** Dr. Yuanhe Yang, tel.: +86-10-6283 6638, E-mail: yhyang@ibcas.ac.cn

This Supplementary Information contains:

Supplementary Notes 1 to 5

Supplementary Tables 1 and 4

Supplementary Figures 1 to 11

Supplementary References

Supplementary Note 1. Characteristics of metagenomic-assembled genomes (MAGs).

We obtained 274 medium quality MAGs (>70% completeness and <10% contamination), among which 125 were high-quality MAGs (>90% completeness and <5% contamination) (Fig. 5a, Supplementary Data 1). There were 34, 94, and 146 MAGs in the surface, subsurface, and permafrost layers, respectively. Most of the genomes constituted, on average, 5.9 ± 0.6%, 10.1 ± 0.6%, and 13.5 ± 0.9% (n = 22, mean ± standard error (SE)) of the total qualified reads in the metagenomes of surface, subsurface, and permafrost layers, respectively (Supplementary Fig. 6). Based on the Genome Taxonomy Database (GTDB r207)[1], a total of 256 bacterial MAGs were mainly annotated as Actinobacteria, Acidobacteria, and Proteobacteria, whereas 18 archaeal MAGs were affiliated to Thermoproteota, Halobacteriota, and Thermoplasmatota (Fig. 5a). At the class level, most MAGs belonged to Thermoleophilia, γ-Proteoribacteria, and UBA4738 (Supplementary Fig. S5), suggesting that these taxa were thriving in the permafrost ecosystem.

Metabolic pathway analysis, based on METABOLIC v4.0[2], unveiled the diverse metabolic potential among genomes (Fig. 5b; Supplementary Data 3). Specifically, it was observed that 272 genomes exhibited the capacity to utilize amino acids, while 198 genomes featured catabolic fatty acid pathways, particularly acyl-CoA dehydrogenase. These results suggested that amino acids and fatty acid may serve as the main substrates for microbial survival in these soils. Moreover, 198 genomes displayed potential for

acetogenesis, and 160 genomes demonstrated a capacity to convert acetate into acetyl-CoA (Fig. 5b), showing that fermentation was an essential metabolic pathway for microbial energy acquisition strategies in permafrost ecosystems[3]. In terms of complex carbon catabolism, 158 genomes contained genes for Chitin degradation, 119 had genes for amylolytic enzymes, while 95 possessed genes for Cellulose degradation (Fig. 5b). Regarding the nitrogen cycle, a substantial number of MAGs exhibited reduction capacity. Specifically, 60 MAGs showed potential for nitrous oxide reduction, 61 MAGs showed competence in nitrite reduction, 60 MAGs featured nitrite reduction to ammonia, and 45 MAGs displayed the capacity for nitrate reduction (Fig. 5b). With regard to other pathways, 273 MAGs had the ability to reduce Fe (Fig. 5b), indicating that Fe reduction may serve as a favorable terminal electron acceptor to fuel microbial anaerobic organic matter degradation[4].

Supplementary Note 2. Metabolic weight scores of each function among whole MAGs.

Based on the metabolic profiles and gene coverage of total 274 MAGs, we calculated metabolic weight scores (MW-score) for each biogeochemical cycling process. High contribution percentage indicates that the microbial group can better represent this function from both gene presence and abundance[44]. The results showed that amino acid utilization (MW-score = 8.9), fermentation (MW-score = 8.7), complex carbon degradation (MW-score = 7.6), and fatty acid degradation (MW-score = 7.3) were the most weighted heterotrophic metabolic pathways at the community level (Fig. 6a). The

higher MW-scores implied that these pathways were essential for microbial metabolism and might play an important role in microbial energy acquisition. In addition, acetate oxidation (MW-score = 5.2), CO oxidation (MW-score = 5), aromatics degradation (MW-score = 4.7), and formate oxidation (MW-score = 5) also contributed highly to the energy acquisition of microorganisms (Fig. 6a). The MW-score results further indicated that sulfur oxidation (MW-score = 4.6) and Fe reduction (MW-score = 5.1) were substantial contributors to the metabolisms of the microbial community (Fig. 6a). Take together, these findings manifested the diverse metabolisms for microbial life in permafrost ecosystems.

Supplementary Note 3. The fractional contribution of microbial taxa to each function for all MAGs.

Based on the results of metabolic weight scores (MW-score) of total 274 MAGs, we calculated the contribution of different taxa to the metabolic weight of each functional pathway. Our results revealed that Actinobacteriota had a high contribution to most of the metabolic pathways at the community level, including most of the complex carbon oxidation, fermentation, and redox reactions involved in the nitrogen, sulfur, and iron metabolic pathways (Fig. 6a). The Actinobacteriota exhibit remarkable metabolic diversity[5] that enables them to contribute highly to the microbial metabolic profiles at the community level. Regarding other taxa, α-Proteobacteria have been documented to have a preference for carbon-rich soils[6,7] and, in line with these observations, we found them to be mainly involved in carbon decomposition processes (including methanol

oxidation, formate oxidation, formaldehyde oxidation, aromatics degradation) and N_2 fixation (Fig. 6a). Acidobacteriota, Chloroflexota, γ-Proteobacteria, and Desulfobacterota were the main microbial taxa making high contributions to redox reactions related to carbon, nitrogen, sulfur, and iron metabolisms (Fig. 6a). Among these microbial groups, Acidobacteria had high contributions to Nitrate reduction, Arsenate reduction, Selenate reduction, Thiosulfate disproportionation and Iron oxidation (Fig. 6a). Chloroflexota were important contributors to nitrite reduction (*nirkS* and *octR*) (Fig. 6a). γ-Proteobacteria were important contributors to microbial oxidation processes including methanotrophy, nitrite ammonification (*nirBD*), Sulfide oxidation, thiosulfate oxidation, iron oxidation and arsenite oxidation (Fig. 6a). Desulfobacterota were mainly important for Wood-Ljungdahl pathway (carbon fixation), nitrite reduction, ammonia oxidation of nitrite (*nrfADH*), and sulfite reduction (Fig. 6a). Overall, these findings demonstrate the high degree of metabolic diversity of microorganisms in permafrost ecosystems.

Supplementary Note 4. The novelty of this study compared with earlier publications.

Although previous studies have explored microbial communities and functional potentials in permafrost soils, this study is innovative in three important respects. First, preceding investigations concerning permafrost microorganisms have predominantly been constrained to the site-specific scale (Supplementary Tables 3-4). Our study provided the first large-scale stratigraphic characteristics of microorganisms in

permafrost regions. To our knowledge, only two studies have focused on microorganisms across the Pan-Arctic permafrost regions by synthesizing sequences data. One of these studies, conducted by Waldrop *et al.*[8], concerned the most highly variable genes in permafrost deposit, and found that these genes were associated with energy metabolism and C-assimilation. The other study, performed by Vishnivetskaya *et al.*[9], revealed that photosynthetic organisms in permafrost deposits were effective members of the re-assembled community after permafrost collapse. In contrast, the data set in our study was derived from systematic measurements along a ~1,000 km transect, and soil samples were collected from both the active layer (surface and subsurface layers) and the permafrost deposit. Based on these systematic measurements, combined with thorough data analysis, our study provided several new findings on permafrost microbes. 1) We observed the lesser effects of environmental variables in permafrost layer comparing to the active layer, suggesting a weaker response of microbes to environment selection in permafrost deposit, which may be ascribed to their survival strategies such as dormancy[10]. 2) We found that genes participating in reduction reactions (e.g., dissimilatory nitrate reduction, denitrification, ferric iron reduction, sulfide reduction, tetrathionate reduction) were enriched in permafrost deposit, implying that microbes colonizing permafrost soils possessed specific metabolic capabilities in spite of the harsh conditions. These findings advanced our understanding of microbial profiles in permafrost regions.

Second, current studies concerning microorganisms were mainly confined to high-

latitude permafrost region, with limited evidence from high-altitude permafrost region (Supplementary Tables 3-4). In this study, we deciphered the microbial diversity, biogeographic patterns from both the taxonomic and phylogenetic points of view, and used the occupancy and specificity analysis method to uncover the specialist species in the surface, subsurface, and permafrost layers. We also employed the null model to explore the underlying assembly mechanisms of microbial communities in each layer. Additionally, we unveiled the variations of microbial functional and metabolic attributes from genetic and genomic points of view. Based on these analyses, this study provides a comprehensive view into microbial communities and functional attributes in the largest permafrost region in the mid- and low latitudes of the world.

Supplementary Note 5. Metabolic weight scores (MW-score) calculation method.

To explore the microbial functional capacity at the community-scale level, we determined the metabolic weight score metric (MW-score) according to the following equation[2]:

$$\mathrm{MW}_{fi} = \frac{\sum_{g=g_1}^{g_n} \mathrm{C}_{g_n} \cdot \mathrm{S}_{f_i}}{\sum_{g=g_1, f=f_1}^{g_n, f_n} \mathrm{C}_{g_n} \cdot \mathrm{S}_{f_n}} \qquad (1)$$

In Eq. (1), the variable MW denotes the MW-score. f_i corresponds to the particular function (f) under consideration, ranked at the i-th (i) position among all functions. g_n represents the n-th genome within the complete set of genomes. f_n signifies the function ranked at the n-th position among all functions. C_g represents the coverage associated with a genome, while S_f signifies the binary state of presence (indicated as 1) or absence (indicated as 0) of a given function within that genome.

We then calculated the percentage contribution of each microbial phylum (the default taxonomic level setting) for each function as follows[2]:

$$\mathrm{Cperc}_{f_{\mathrm{i}}p_{\mathrm{i}}} = \left(\frac{\sum_{g=g_{\mathrm{k}}}^{g_{\mathrm{i}}} \mathrm{C}_{g_{\mathrm{n}}} \cdot \mathrm{S}_{f_{\mathrm{i}}}}{\sum_{g=g_{1},f=f_{1}}^{g_{\mathrm{n}},f_{\mathrm{n}}} \mathrm{C}_{g_{\mathrm{n}}} \cdot \mathrm{S}_{f_{\mathrm{n}}}} \bigg/ \frac{\sum_{g=g_{1}}^{g_{\mathrm{n}}} \mathrm{C}_{g_{\mathrm{n}}} \cdot \mathrm{S}_{f_{\mathrm{i}}}}{\sum_{g=g_{1},f=f_{1}}^{g_{\mathrm{n}},f_{\mathrm{n}}} \mathrm{C}_{g_{\mathrm{n}}} \cdot \mathrm{S}_{f_{\mathrm{n}}}} \right) \times 100\% \qquad (2)$$

In Eq. (2), Cprec denotes the percentage contribution of a microbial group to the MW-score. p_j is the specific group (p) under investigation, ranked at the j-th (j) position among all groups. The variables g_k and g_l represent genomes ranked at the k-th (k) and l-th (l) positions, respectively, among all genomes. The additional notation $g_k...g_l \in p_j$ signifies that all genomes falling within this range are encompassed within the studied group p_j. Both the MW-score and percentage contribution are determined via METABOLIC v4.0[2].

Table S1. Site information about the vegetation, coordinate location, and climatic characteristics. Soil samples were collected at 24 sites along a ~1,000 km permafrost transect on the plateau. At two of these sites, there were problems obtaining a sufficient DNA yield during DNA extraction and so only samples from 22 sites were processed in this study. AI, aridity index, determined by dividing mean annual precipitation by mean annual potential evapotranspiration[11], which was retrieved from the CGIAR-CSI Global-Aridity and Global-PET database (http://www.cgiar-csi.org). NDVI, Normalized Difference Vegetation Index. NDVI data were obtained from the Moderate Resolution Imaging Spectroradiometer (MODIS) aboard NASA's Terra satellites (http://neo.sci.gsfc.nasa.gov/) with ~1 km resolution for every 16-day interval between July to August in 2016 (when soil sampling was conducted).

Site No.	Vegetation type	Latitude (°N)	Longitude (°E)	AI	NDVI
S01	Swamp meadow	34.14	97.67	0.70	0.53
S02	Swamp meadow	34.26	97.85	0.63	0.48
S03	Swamp meadow	34.44	97.94	0.47	0.46
S04	Swamp meadow	34.02	97.54	0.61	0.44
S05	Swamp meadow	35.06	98.69	0.52	0.40
S06	Swamp meadow	34.80	99.06	0.68	0.55
S07	Swamp meadow	34.20	99.24	0.57	0.61
S08	Swamp meadow	34.54	99.15	0.58	0.56
S09	Swamp meadow	34.67	99.12	0.63	0.52
S10	Alpine meadow	35.49	99.5	0.57	0.61
S11	Alpine meadow	38.58	98.17	0.32	0.34
S12	Swamp meadow	38.32	98.27	0.37	0.44
S13	Swamp meadow	37.85	98.44	0.32	0.48
S14	Alpine meadow	33.26	91.86	0.39	0.4
S15	Alpine meadow	32.58	91.86	0.52	0.55
S16	Swamp meadow	32.96	91.99	0.52	0.26
S17	Alpine meadow	34.30	92.52	0.26	0.22
S18	Alpine meadow	34.69	92.91	0.37	0.35
S19	Alpine meadow	34.69	92.91	0.37	0.41
S20	Alpine meadow	34.86	92.94	0.27	0.39
S21	Alpine steppe	35.18	93.04	0.28	0.19
S22	Alpine steppe	35.62	94.07	0.30	0.12

Table S2. Results of three non-parametric multivariate statistical approaches employed to test the differences in microbial taxonomy, phylogeny, functional genes and metagenome-assembled genomes (MAGs) with soil depth. Variations of the microbial taxonomic, phylogenetic, functional genetic, and genomic composition are determined by the Bray-Curtis distance. The three non-parametric multivariate statistical approaches are permutational multivariate analysis of variance (Adonis), analysis of similarity (ANOSIM), and multi-response permutation procedures (MRPP). $n = 22$. P values less than 0.05 are in bold.

Item	Adonis		ANOSIM		MRPP	
	F	*P*	R	*P*	δ	*P*
Taxonomy	6.07	**0.001**	0.39	**0.001**	0.71	**0.001**
Phylogeny	7.17	**0.001**	0.32	**0.001**	0.24	**0.001**
Functional genes	8.97	**0.001**	0.39	**0.001**	0.12	**0.001**
MAGs	10.16	**0.001**	0.44	**0.001**	0.49	**0.001**

Table S3. **Summary of studies concerning permafrost microorganisms based on meta-omics data.** "Measured" in the data source column means that data are measured with the same procedures, while "Synthesized" indicates that data are collected from different studies/projects. MG, metagenomics; MT, metatranscriptomics; MP, metaproteomics.

References	Region	No. of sites	Data source	Methods
Mackelprang *et al.*[12]	Pan-Arctic	1	Measured	Amplicon, MG, qPCR
Tveit *et al.*[13]	Pan-Arctic	1	Measured	MT, MG
Xue *et al.*[14]	Pan-Arctic	1	Measured	Amplicon, GeoChip,
Johnston *et al.*[15]	Pan-Arctic	1	Measured	Amplicon, MG
Wu *et al.*[16]	Pan-Arctic	1	Measured	Amplicon, GeoChip,
Mondav *et al.*[17]	Pan-Arctic	1	Measured	Amplicon, MG
McCalley *et al.*[18]	Pan-Arctic	1	Measured	Amplicon, MG
Hultman *et al.*[19]	Pan-Arctic	1	Measured	Amplicon, MG, MT, MP
Singleton *et al.*[20]	Pan-Arctic	1	Measured	MG, MT
Woodcroft *et al.*[21]	Pan-Arctic	1	Measured	Amplicon, MG, MT
Yergeau *et al.*[22]	Pan-Arctic	1	Measured	16S, qPCR
Tveit *et al.*[23]	Pan-Arctic	2	Measured	MG, MT
Taş *et al.*[24]	Pan-Arctic	1	Measured	Amplicon, MG
Geisen *et al.*[25]	Pan-Arctic	1	Measured	MT
Mackelprang *et al.*[26]	Pan-Arctic	1	Measured	Amplicon, MG
Müller *et al.*[27]	Pan-Arctic	1	Measured	Amplicon, MG
Taş *et al.*[28]	Pan-Arctic	1	Measured	Amplicon, MG
Wu *et al.*[29]	Pan-Arctic	1	Measured	Amplicon, MG
Wu *et al.*[30]	Pan-Arctic	2	Measured	Amplicon, MG
Waldrop *et al.*[8]	Pan-Arctic	18	Synthesized	MG
Vishnivetskaya *et al.*[9]	Pan-Arctic	11	Synthesized	Amplicon, MG
Tang *et al.*[31]	Tibetan Plateau	4	Measured	Amplicon, qPCR
This study	Tibetan Plateau	22	Measured	Amplicon, MG

Table S4: **Summary of studies related to the microbial assemblage mechanisms in permafrost deposits.**

References	Region	No. of sites	Sample source
Hu *et al.*[32]	Tibetan Plateau	1	Intact permafrost
Bottos *et al.*[33]	Pan-Arctic	1	Intact permafrost
Doherty *et al.*[34]	Pan-Arctic	1	Thawed permafrost
Mondav *et al.*[35]	Pan-Arctic	1	Active layer of thawing permafrost landscapes
Feng *et al.*[36]	Pan-Arctic	1	Active layer of thawing permafrost landscapes
Wu *et al.*[37]	Tibetan Plateau	1	Active layer of thawing permafrost landscapes
Wu *et al.*[16]	Pan-Arctic	1	Active layer of thawing permafrost landscapes
This study	Tibetan Plateau	22	Active layer and intact permafrost

Figure S1. Profile of microbial abundance and composition of dominant phyla across the Tibetan alpine permafrost region. **a** The number of the amplicon sequence variants (ASVs) and corresponding percentage (denoted in parentheses) of dominant phyla for all soil samples among three soil layers. **b** The relative abundance of the dominant phyla for all soil samples among three soil layers. **c** The relative abundance of the dominant phyla in each soil layers. SUR, surface layer; SUB, subsurface layer; PL, permafrost layer.

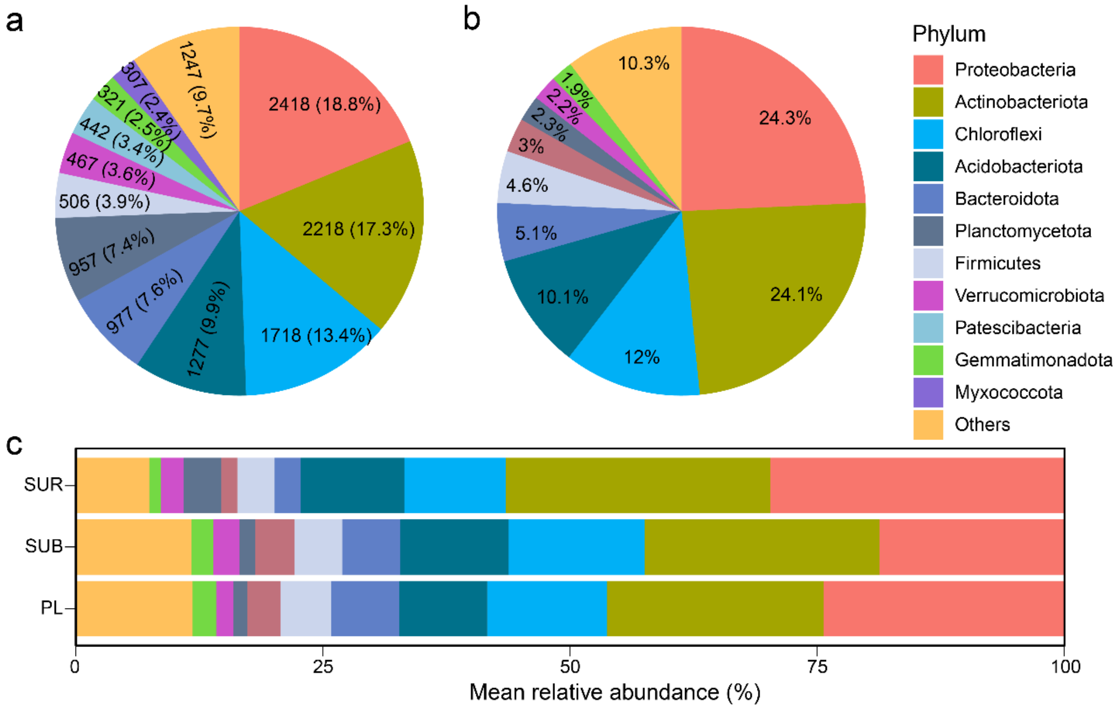

Figure S2. Clustering analysis for reducing the environmental variables redundancy based on the varclus procedure in the Hmisc[38] R package. Variables with a high correlation (Spearman's $\rho^2 > 0.7$) were removed to avoid redundancy[39]. MAT, mean annual air temperature; AI, aridity index; NDVI, Normalized Difference Vegetation Index; SR, plant species richness; HF, human footprint index; SOC, soil organic carbon; DOC, dissolved organic carbon; DON, dissolved organic nitrogen; LCP1, labile carbon pool I (mainly polysaccharides); LCP2, labile carbon pool II (mostly cellulose); RCP, recalcitrant carbon pool.

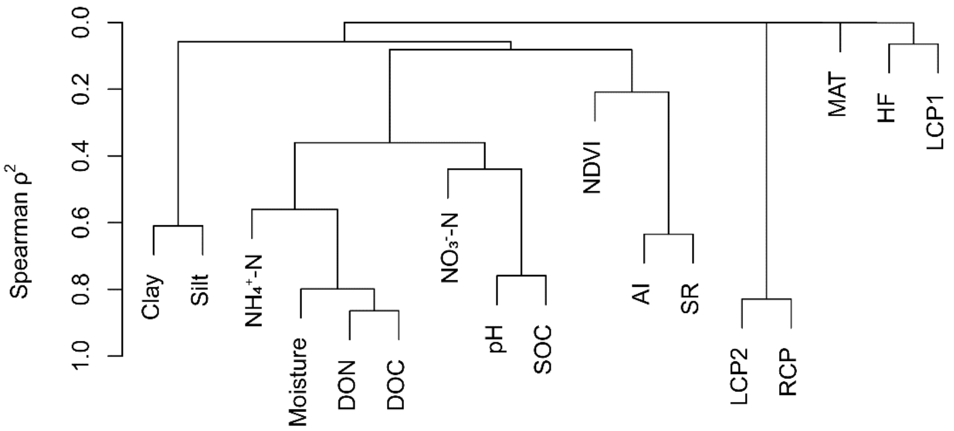

Figure S3. The number of diurnal freeze-thaw events within one year over soil depth on the Tibetan Plateau. A diurnal freezing-thawing event, as defined by Baker and Ruschy[40], is recorded when the daily minimum soil temperature drops below 0°C, and the daily maximum soil temperature reaches 0°C or higher. Soil temperature data were derived from seventeen sites on the Tibetan Plateau and were recorded by Yang *et al.*[41], Wei *et al.*[42], and our research group, respectively.

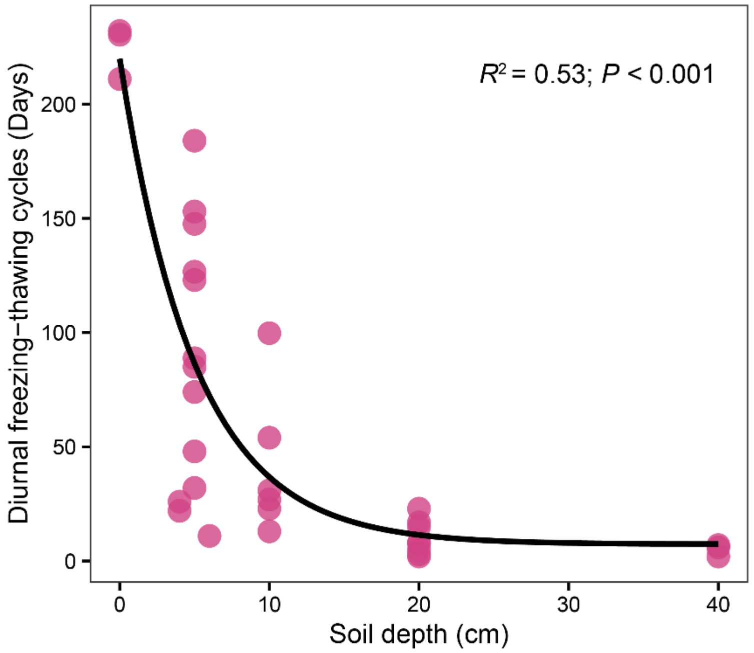

Figure S4. **Profile of the functional genes among various soil layers**. **a** The composition of the functional genes for the KEGG pathway. **b** Results of the principal component analysis using the functional genes (n = 22). **c** Variations of functional gene composition among three soil layers (n = 231). Gene compositional variations are determined based on the Bray-Curtis distance. Different lowercase letters in the box plots denote significant differences among three soil layers (determined by two-sided pairwise Wilcoxon test, $P < 0.05$). Central line and whiskers in each box represent the median and 1.5 times the interquartile range, respectively. Boxes indicate the interquartile range between 25th and 75th percentile. Single points are outliers. SUR, surface layer; SUB, subsurface layer; PL, permafrost layer. Source data are provided as a Source Data file.

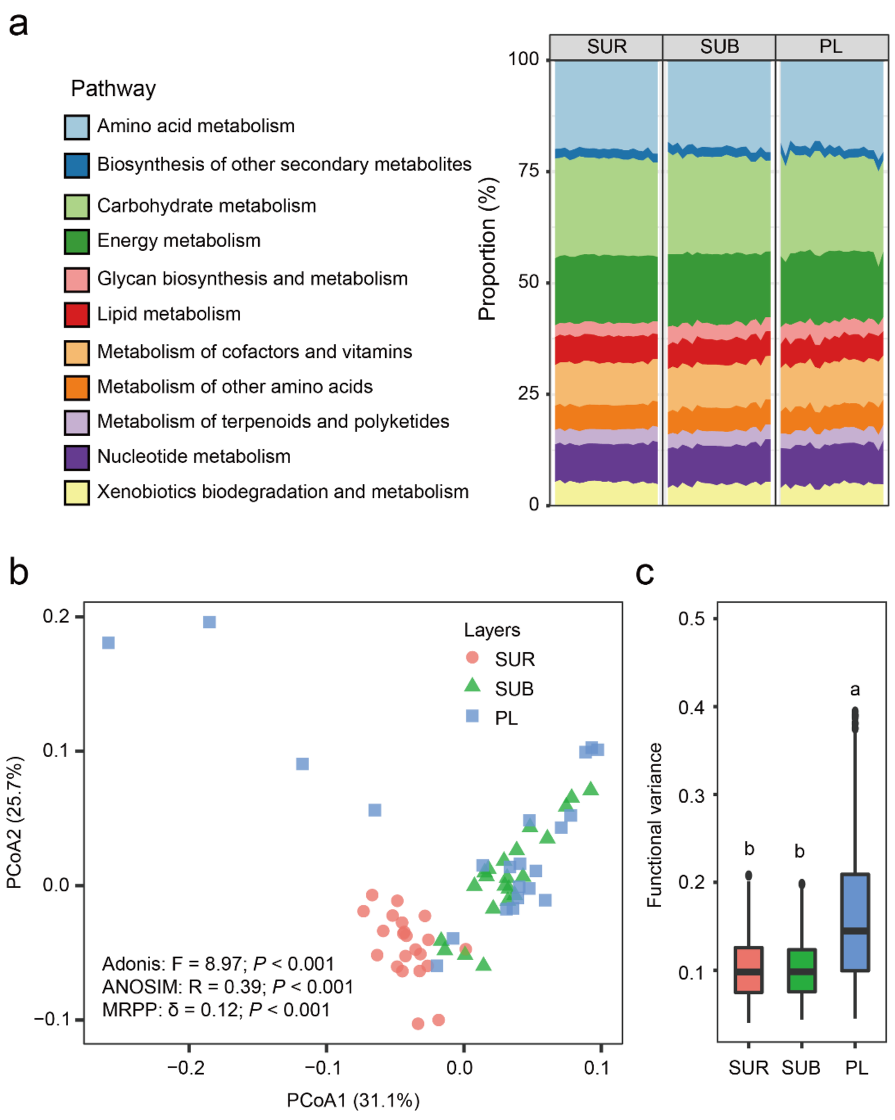
a
Pathway
Amino acid metabolism
Biosynthesis of other secondary metabolites
Carbohydrate metabolism
Energy metabolism
Glycan biosynthesis and metabolism
Lipid metabolism
Metabolism of cofactors and vitamins
Metabolism of other amino acids
Metabolism of terpenoids and polyketides
Nucleotide metabolism
Xenobiotics biodegradation and metabolism
Proportion (%)
100
75
50
25
0
SUR
SUB
PL
b
Layers
SUR
SUB
PL
Adonis: F = 8.97; P < 0.001
ANOSIM: R = 0.39; P < 0.001
MRPP: δ = 0.12; P < 0.001
PCoA2 (25.7%)
PCoA1 (31.1%)
0.2
0.1
0.0
−0.1
−0.2
−0.1
0.0
0.1
c
Functional variance
0.5
0.4
0.3
0.2
0.1
a
b
b
SUR
SUB
PL

Figure S5. The taxonomic distribution of the total 274 metagenome-assembled genomes (MAGs) at the class level. Source data are provided as a Source Data file.

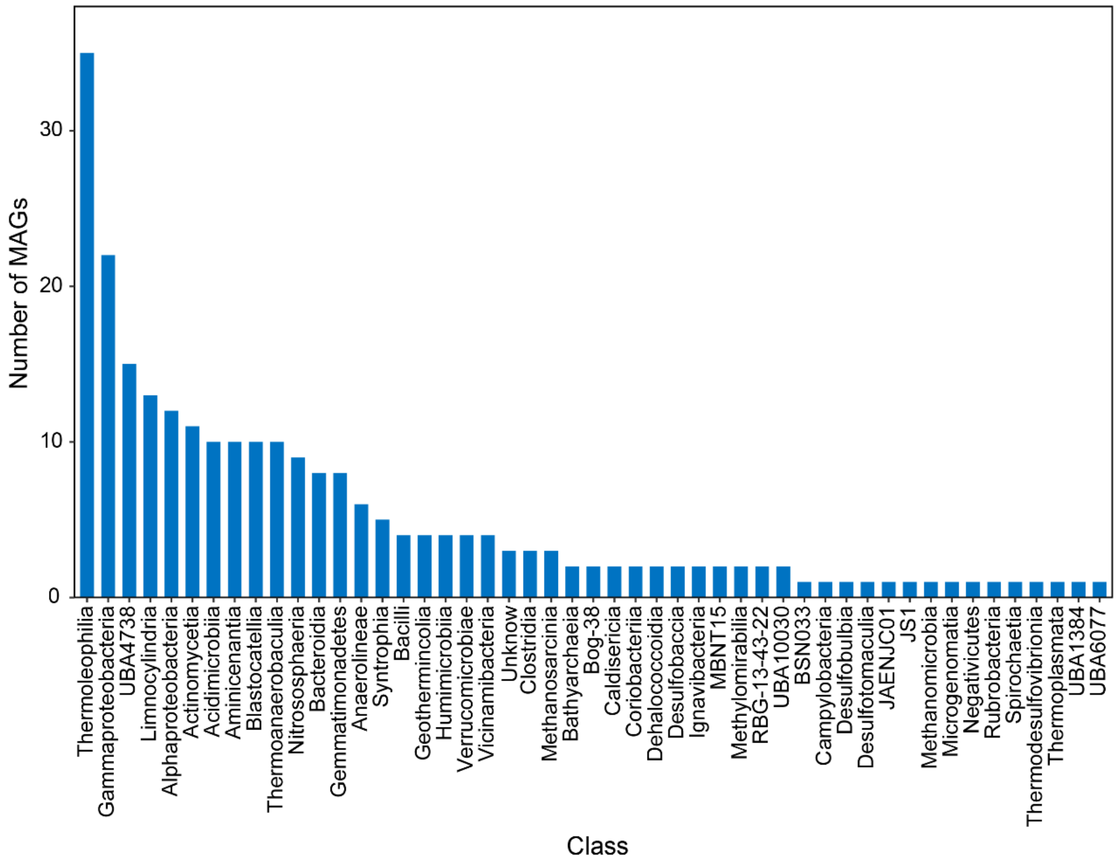

Figure S6. The difference in sequencing and metagenome-assembled genomes (MAGs) characteristics among three soil layers, determined by pairwise Wilcoxon test. **a** The difference in sequencing depth among three soil layers (*n* = 22 in each soil layer, two-sided pairwise Wilcoxon test). **b-d** The differences in completeness (**b**) (SUR: *n* = 34; SUB: *n* = 94; PL: *n* = 146), contamination (**c**) (SUR: *n* = 34; SUB: *n* = 94; PL: *n* = 146), and the recovery rate (**d**) (*n* = 22 in each soil layer) of MAGs among three soil layers (Two-sided Wilcoxon test). Different lowercase letters in box plots indicate significant differences among three soil layers ($P < 0.05$). Central line and whiskers in each box represent the median and 1.5 times the interquartile range, respectively. Boxes indicate the interquartile range between 25th and 75th percentile. Single points are outliers. SUR, surface layer; SUB, subsurface layer; PL, permafrost layer. Source data are provided as a Source Data file.

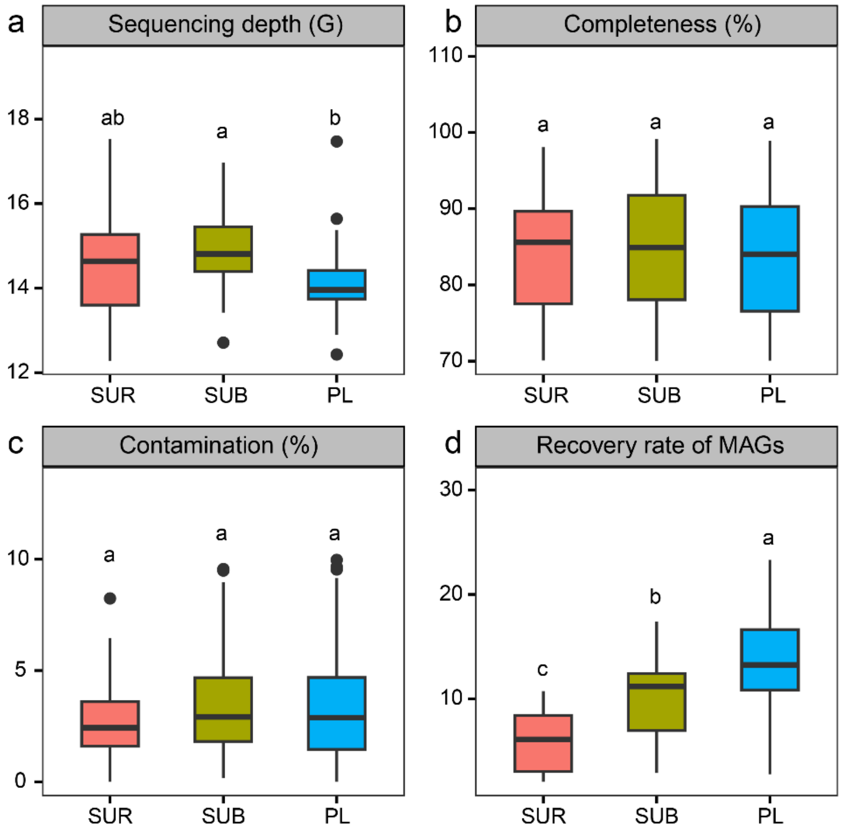

Figure S7. Differences in environmental variables among three soil layers. Variations of clay (**a**), silt (**b**), pH (**c**), soil moisture (**d**), soil organic carbon (SOC) (**e**), dissolved organic carbon (DOC) (**f**), labile carbon pool I (LCP1, mainly polysaccharides) (**g**), labile carbon pool II (LCP2, mostly cellulose) (**h**), recalcitrant carbon pool (RCP) (**i**), NH_4^+-N (**j**), NO_3^--N (**k**), and dissolved organic nitrogen (DON) (**l**) among three soil layers. Different lowercase letters in box plots indicate significant differences, which were determined by two-sided pairwise Wilcoxon test (n = 22). Different lowercase letters in bar plots indicate significant differences among three soil layers ($P < 0.05$). The error bars indicate the standard error of each variables. Soil moisture is defined as the percentage of water present in soil mass by its weight. SUR, surface layer; SUB, subsurface layer; PL, permafrost layer. Soil texture data were obtained from Mao *et al.*[43], the remaining variables were determined by ourselves. Source data are provided as a Source Data file.

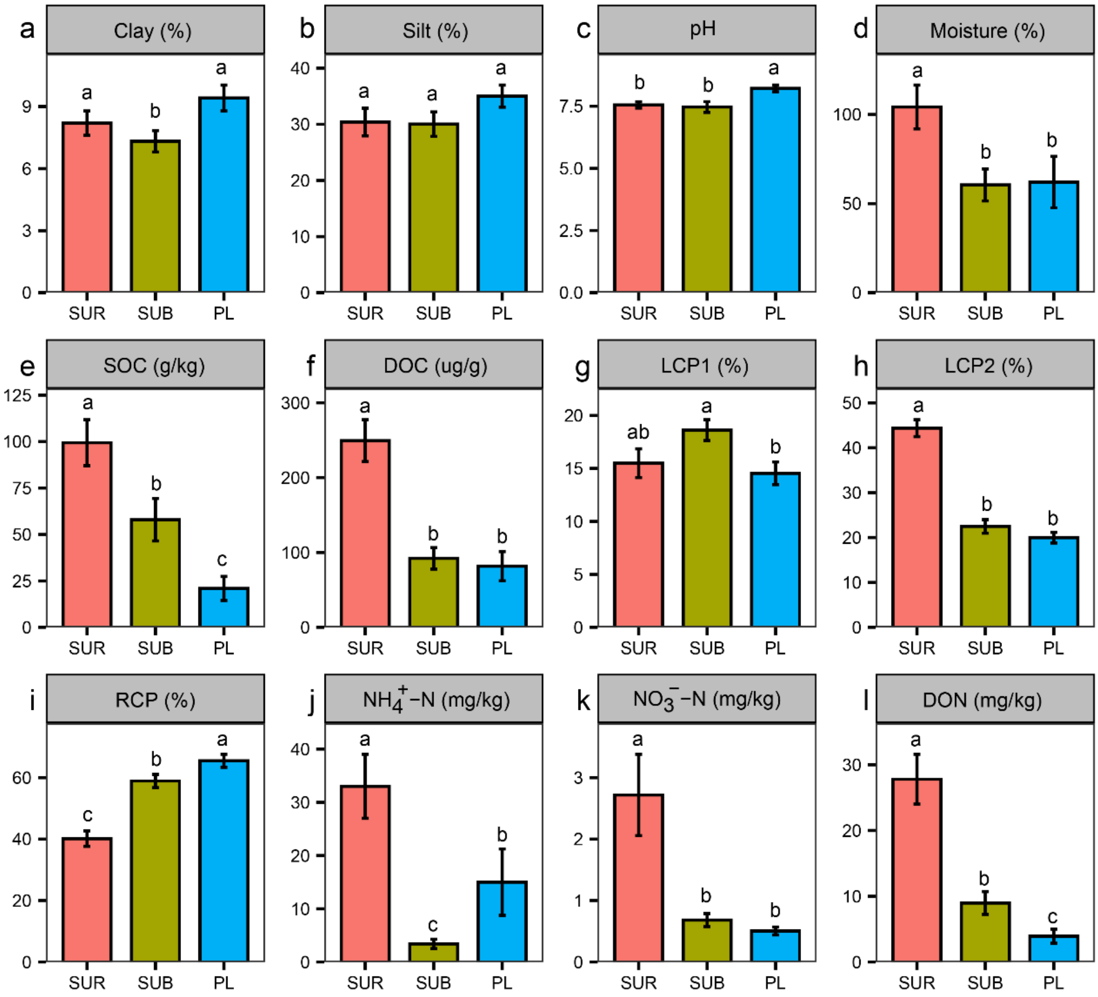

Figure S8. The presence of genes encoding fermentation on the metagenome-assembled genomes (MAGs) among three soil layers. **a** Surface layer (SUR). **b** Subsurface layer (SUB). **c** Permafrost layer (PL).

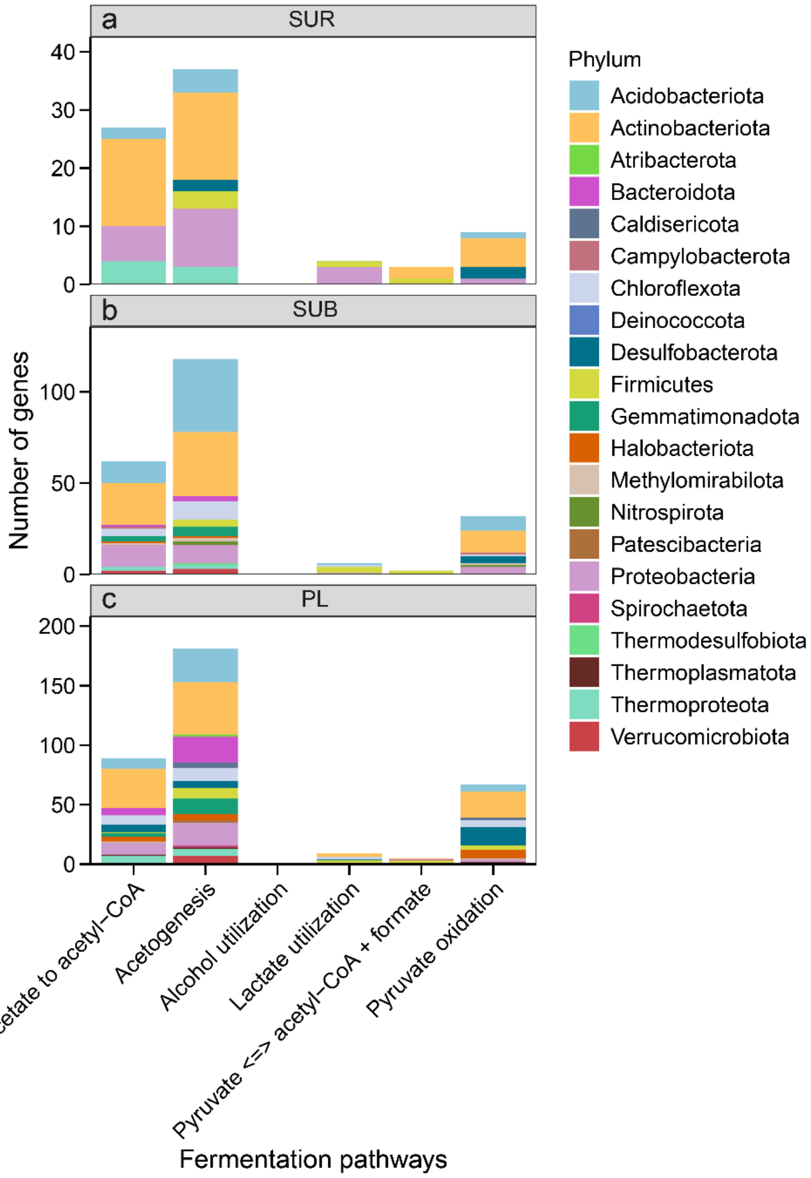

Figure S9. The relationships between biogeochemical processes and gene relative abundance. a-i Linear regression relationships between cumulative CO_2 release and the relative abundance of genes encoding carbon degradation. **j-l** The relationships between CH_4 production and the relative abundance of genes involved in methane production and oxidation. **m-o** The relationships of nitrogen mineralization and nitrification rate with the relative abundance of genes participated in nitrogen cycling. Cumulative CO_2 release in permafrost soils was measured via 400-day incubation at 5°C by Qin *et al.*[44]. CH_4 production potentials of subsurface and permafrost soils was measured through 24 h incubation at 4°C using the subsurface and permafrost soil samples by Song *et al.*[45]. Nitrogen cycling processes of three soil layers were measured via 24 h incubation at 5°C using ^{15}N labeling by Mao *et al.*[43]. All these biogeochemical processes were measured using the same soil samples as in this study. Shaded area shows the 95% confidence interval.

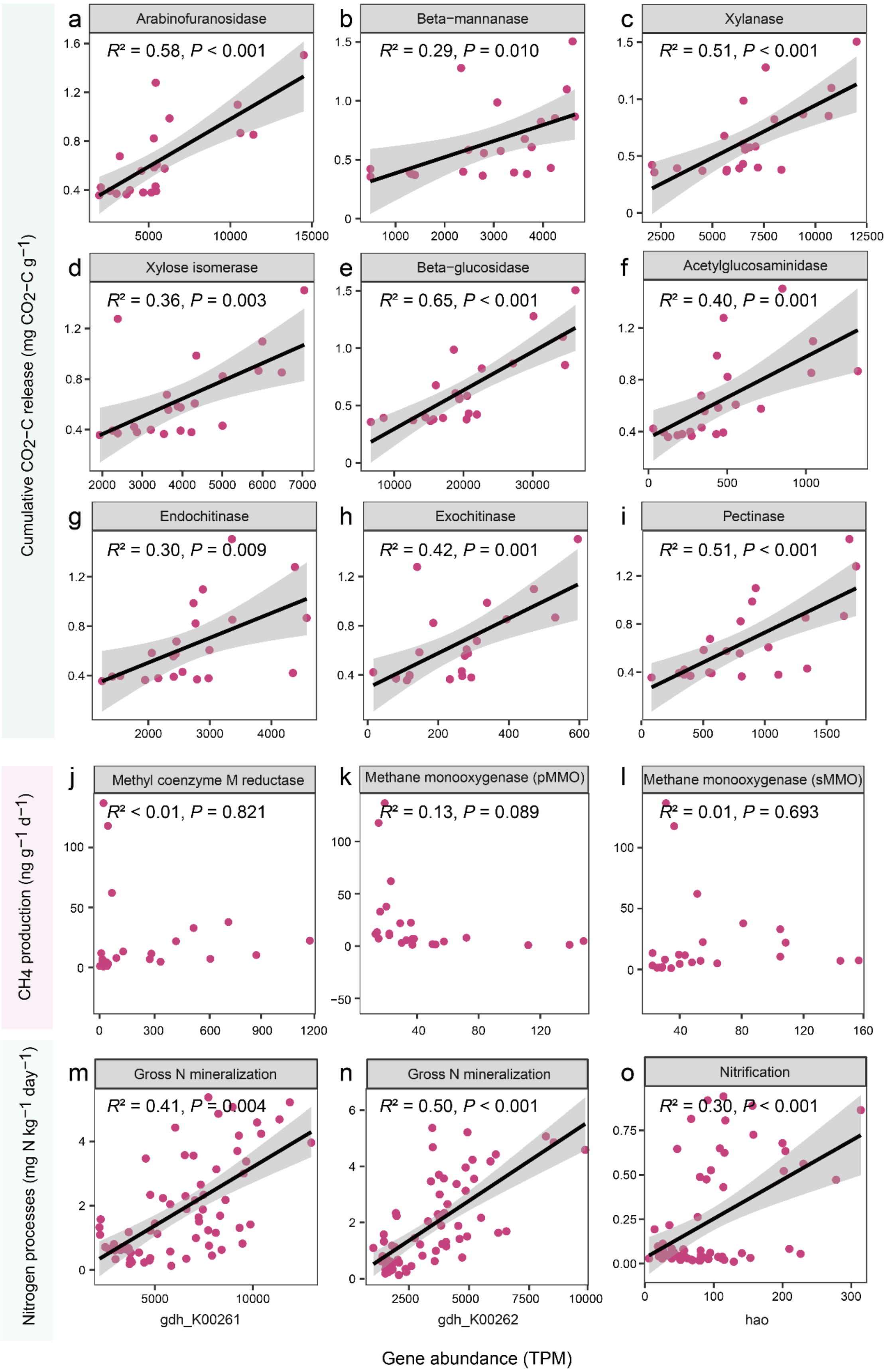

Cumulative CO2-C release (mg CO2-C g−1)
a Arabinofuranosidase
R² = 0.58, P < 0.001
b Beta−mannanase
R² = 0.29, P = 0.010
c Xylanase
R² = 0.51, P < 0.001
d Xylose isomerase
R² = 0.36, P = 0.003
e Beta−glucosidase
R² = 0.65, P < 0.001
f Acetylglucosaminidase
R² = 0.40, P = 0.001
g Endochitinase
R² = 0.30, P = 0.009
h Exochitinase
R² = 0.42, P = 0.001
i Pectinase
R² = 0.51, P < 0.001
CH4 production (ng g−1 d−1)
j Methyl coenzyme M reductase
R² < 0.01, P = 0.821
k Methane monooxygenase (pMMO)
R² = 0.13, P = 0.089
l Methane monooxygenase (sMMO)
R² = 0.01, P = 0.693
Nitrogen processes (mg N kg−1 day−1)
m Gross N mineralization
R² = 0.41, P = 0.004
n Gross N mineralization
R² = 0.50, P < 0.001
o Nitrification
R² = 0.30, P < 0.001
gdh_K00261
gdh_K00262
hao
Gene abundance (TPM)

Figure S10. The comparison of human footprint (HF) index for our sampling sites and several typical cities (Lhasa, Golmud, Yushu, Nagqu, Madoi, Qilian, and Xining) on the Tibetan Plateau. HF data were obtained from National Tibetan Plateau Data Center (https://doi.org/10.11922/sciencedb.933). A higher HF value indicates more disturbance, and vice versa. Different lowercase letters indicate significant differences ($P < 0.05$), which were determined by two-sided Wilcoxon test (Sampling sites: $n = 22$; Cities: $n = 7$). Central line and whiskers in each box represent the median and 1.5 times the interquartile range, respectively. Boxes indicate the interquartile range between 25th and 75th percentile. Single points are outliers. Source data are provided as a Source Data file.

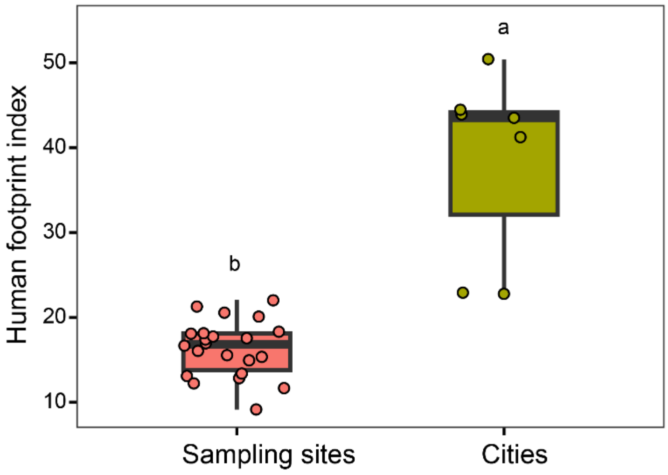

Figure S11. A general framework for bioinformatic analysis involved in this study. Amplicon data were processed using the UNOISE method, and the taxonomic and phylogenetic information was obtained based on the amplicon sequencing. Metagenomic data were first assembled to contigs, genes were predicted and annotated at the contigs level. The assembled contigs were then further binned to metagenome-assembled genomes (MAGs), and the taxonomic and metabolic profiles were annotated using GTDB-tk v2.1.1[1] and METABOLIC v4.0[2], respectively. This diagram was drawn by Microsoft PowerPoint 2016 (Microsoft Corporation).

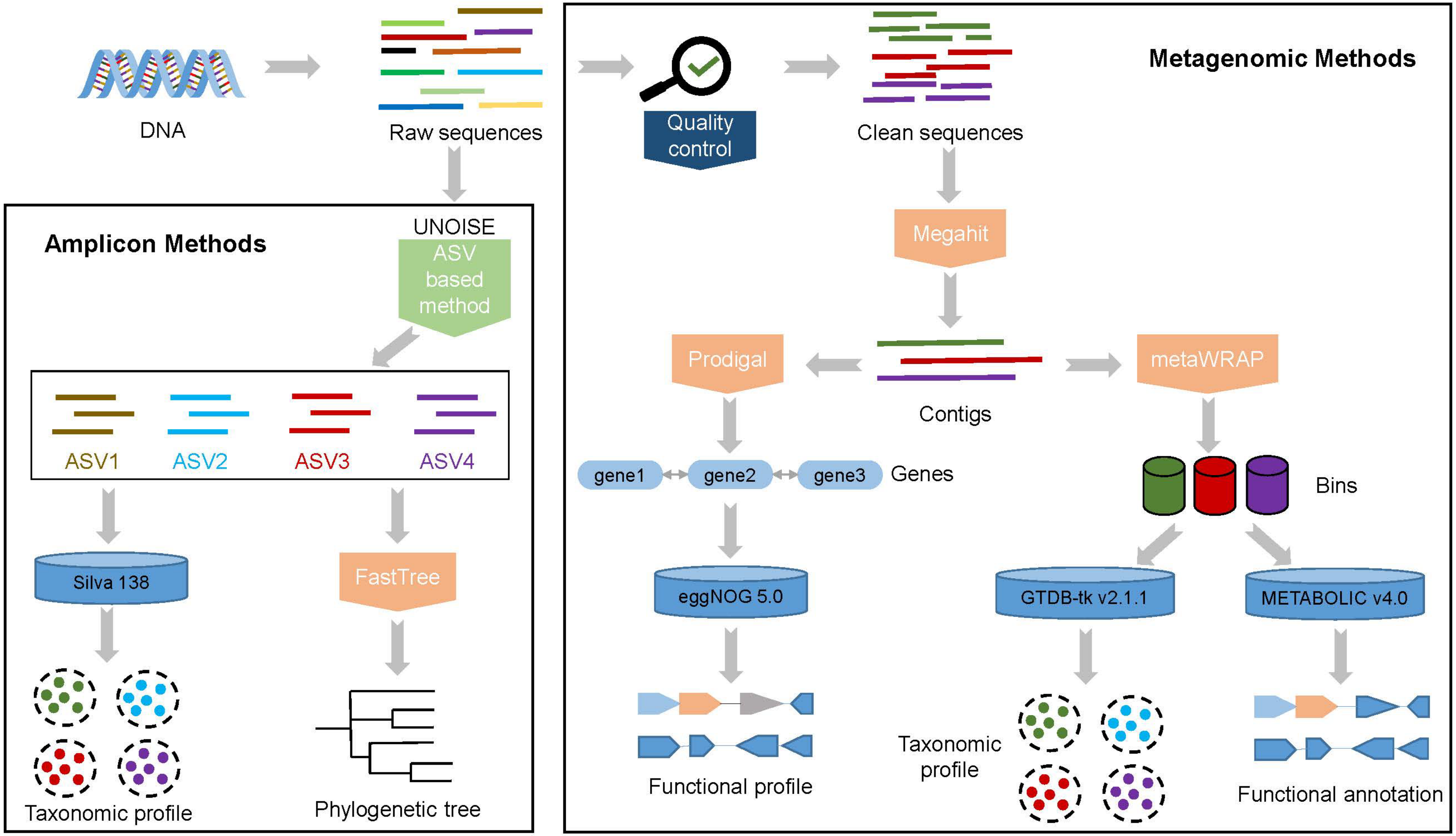
DNA
Raw sequences
Quality control
Clean sequences
Metagenomic Methods
Amplicon Methods
UNOISE
ASV based method
ASV1
ASV2
ASV3
ASV4
Silva 138
FastTree
Taxonomic profile
Phylogenetic tree
Megahit
Prodigal
Contigs
metaWRAP
gene1
gene2
gene3
Genes
Bins
eggNOG 5.0
GTDB-tk v2.1.1
METABOLIC v4.0
Functional profile
Taxonomic profile
Functional annotation

Supplementary References

1. Chaumeil, P.A., Mussig, A. J., Hugenholtz, P. & Parks, D. H. GTDB-Tk: a toolkit to classify genomes with the Genome Taxonomy Database. *Method. Biochem. Anal.* **36**, 1925–1927 (2020).
2. Zhou, Z. *et al.* METABOLIC: high-throughput profiling of microbial genomes for functional traits, metabolism, biogeochemistry, and community-scale functional networks. *Microbiome* **10**, 33 (2022).
3. Altshuler, I., Goordial, J. & Whyte, L. G. Microbial Life in Permafrost. in *Psychrophiles: From Biodiversity to Biotechnology* (ed. Margesin, R.) 153–179 (Springer International Publishing, 2017).
4. Lipson, D. A., Jha, M., Raab, T. K. & Oechel, W. C. Reduction of iron (III) and humic substances plays a major role in anaerobic respiration in an Arctic peat soil. *J. Geophys. Res.: Biogeosci.* **115**, (2010).
5. Bull, A. T. Actinobacteria of the Extremobiosphere. in *Extremophiles Handbook* (ed. Horikoshi, K.) 1203–1240 (Springer Japan, 2011).
6. Nemergut, D. R., Cleveland, C. C., Wieder, W. R., Washenberger, C. L. & Townsend, A. R. Plot-scale manipulations of organic matter inputs to soils correlate with shifts in microbial community composition in a lowland tropical rain forest. *Soil Biol. Biochem.* **42**, 2153–2160 (2010).
7. Goldfarb, K. *et al.* Differential growth responses of soil bacterial taxa to carbon substrates of varying chemical recalcitrance. *Front. Microbiol.* **2**, (2011).
8. Waldrop, M. P. *et al.* Permafrost microbial communities and functional genes are

structured by latitudinal and soil geochemical gradients. *ISME J.* **17**, 1224–1235 (2023).

9. Vishnivetskaya, T. A. *et al.* Insights into community of photosynthetic microorganisms from permafrost. *FEMS Microbiol. Ecol.* **96**, fiaa229 (2020).
10. Nemergut, D. R. *et al.* Patterns and processes of microbial community assembly. *Microbiol. Mol. Biol. Rev.* **77**, 342–356 (2013).
11. Zomer, R. J., Trabucco, A., Bossio, D. A. & Verchot, L. V. Climate change mitigation: A spatial analysis of global land suitability for clean development mechanism afforestation and reforestation. *Agric. Ecosyst. Environ.* **126**, 67–80 (2008).
12. Mackelprang, R. *et al.* Metagenomic analysis of a permafrost microbial community reveals a rapid response to thaw. *Nature* **480**, 368–371 (2011).
13. Tveit, A. T., Urich, T., Frenzel, P. & Svenning, M. M. Metabolic and trophic interactions modulate methane production by Arctic peat microbiota in response to warming. *Proc. Natl. Acad. Sci. USA* **112**, E2507–E2516 (2015).
14. Xue, K. *et al.* Tundra soil carbon is vulnerable to rapid microbial decomposition under climate warming. *Nat. Clim. Chang.* **6**, 595–600 (2016).
15. Johnston, E. R. *et al.* Responses of tundra soil microbial communities to half a decade of experimental warming at two critical depths. *Proc. Natl Acad. Sci. USA* **116**, 15096–15105 (2019).
16. Wu, L. *et al.* Permafrost thaw with warming reduces microbial metabolic capacities in subsurface soils. *Mol. Ecol.* **31**, 1403–1415 (2022).

17. Mondav, R. *et al.* Discovery of a novel methanogen prevalent in thawing permafrost. *Nat. Commun.* **5**, 3212 (2014).
18. McCalley, C. K. *et al.* Methane dynamics regulated by microbial community response to permafrost thaw. *Nature* **514**, 478–481 (2014).
19. Hultman, J. *et al.* Multi-omics of permafrost, active layer and thermokarst bog soil microbiomes. *Nature* **521**, 208–212 (2015).
20. Singleton, C. M. *et al.* Methanotrophy across a natural permafrost thaw environment. *ISME J.* **12**, 2544–2558 (2018).
21. Woodcroft, B. J. *et al.* Genome-centric view of carbon processing in thawing permafrost. *Nature* **560**, 49–54 (2018).
22. Yergeau, E., Hogues, H., Whyte, L. G. & Greer, C. W. The functional potential of high Arctic permafrost revealed by metagenomic sequencing, qPCR and microarray analyses. *ISME J.* **4**, 1206–1214 (2010).
23. Tveit, A., Schwacke, R., Svenning, M. M. & Urich, T. Organic carbon transformations in high-Arctic peat soils: key functions and microorganisms. *ISME J.* **7**, 299–311 (2013).
24. Taş, N. *et al.* Impact of fire on active layer and permafrost microbial communities and metagenomes in an upland Alaskan boreal forest. *ISME J.* **8**, 1904–1919 (2014).
25. Geisen, S. *et al.* Metatranscriptomic census of active protists in soils. *ISME J.* **9**, 2178–2190 (2015).
26. Mackelprang, R. *et al.* Microbial survival strategies in ancient permafrost: insights from metagenomics. *ISME J.* **11**, 2305–2318 (2017).

27. Müller, O. *et al.* Disentangling the complexity of permafrost soil by using high resolution profiling of microbial community composition, key functions and respiration rates. *Environ. Microbiol.* **20**, 4328–4342 (2018).

28. Taş, N. *et al.* Landscape topography structures the soil microbiome in arctic polygonal tundra. *Nat. Commun.* **9**, 777 (2018).

29. Wu, X. *et al.* Comparative metagenomics of the active layer and permafrost from low-carbon soil in the Canadian High Arctic. *Environ. Sci. Technol.* **55**, 12683–12693 (2021).

30. Wu, X. *et al.* Microbial life in 25-m-deep boreholes in ancient permafrost illuminated by metagenomics. *Environ. Microbiome* **18**, 1–19 (2023).

31. Tang, X. *et al.* Changing microbiome community structure and functional potential during permafrost thawing on the Tibetan Plateau. *FEMS Microbiol. Ecol.* **99**, fiad117 (2023).

32. Hu, W. *et al.* Relative roles of deterministic and stochastic processes in driving the vertical distribution of bacterial communities in a permafrost core from the Qinghai-Tibet Plateau, China. *PLoS One* **10**, e0145747 (2015).

33. Bottos, E. M. *et al.* Dispersal limitation and thermodynamic constraints govern spatial structure of permafrost microbial communities. *FEMS Microbiol. Ecol.* **94**, fiy110 (2018).

34. Doherty, S. J. *et al.* The transition from stochastic to deterministic bacterial community assembly during permafrost thaw succession. *Front. Microbiol.* **11**, 596589 (2020).

35. Mondav, R. *et al.* Microbial network, phylogenetic diversity and community membership in the active layer across a permafrost thaw gradient. *Environ. Microbiol.* **19**, 3201–3218 (2017).

36. Feng, J. *et al.* Warming-induced permafrost thaw exacerbates tundra soil carbon decomposition mediated by microbial community. *Microbiome* **8**, 3 (2020).

37. Wu, M.H. *et al.* Soil microbial distribution and assembly are related to vegetation biomass in the alpine permafrost regions of the Qinghai-Tibet Plateau. *Sci. Total Environ.* **834**, 155259 (2022).

38. Jr, F. E. H. *Hmisc: Harrell Miscellaneous*. R package version 4.7-0 (2022).

39. Wang, X. *et al.* Habitat-specific patterns and drivers of bacterial β-diversity in China's drylands. *ISME J.* **11**, 1345 (2017).

40. Baker, D. G. & Ruschy, D. L. Calculated and measured air and soil freeze–thaw frequencies. *J. Appl. Meteorol.* **34**, 2197–2205 (1995).

41. Yang, K., Yang, K. & Su, B. Time-lapse observation dataset of soil temperature and humidity on the Tibetan Plateau (2008-2016). (National Tibetan Plateau Data Center, 2019)

42. Wei, D. *et al.* Plant uptake of CO2 outpaces losses from permafrost and plant respiration on the Tibetan Plateau. *Proceedings of the National Academy of Sciences* **118**, e2015283118 (2021).

43. Mao, C. *et al.* Permafrost nitrogen status and its determinants on the Tibetan Plateau. *Glob. Change Biol.* **26**, 5290–5302 (2020).

44. Qin, S. *et al.* Temperature sensitivity of permafrost carbon release mediated by

mineral and microbial properties. *Sci. Adv.* **7**, eabe3596 (2021).

45. Song, Y. *et al.* Methanogenic community, CH_4 production potential and its determinants in the active layer and permafrost deposits on the Tibetan Plateau. *Environ. Sci. Technol.* **55**, 11412–11423 (2021).
