# Supplementary material for: Metagenomic insights into microbial community structure and metabolism in alpine permafrost on the Tibetan Plateau
Source: Nat Commun. 2024 Jul 14;15:5920. doi: 10.1038/s41467-024-50276-2 (PMC11247091; doi:10.1038/s41467-024-50276-2)
Supplement: Supplementary file 3 — Description of Additional Supplementary Files [file 41467_2024_50276_MOESM3_ESM.pdf]

Description of Additional Supplementary Files:

Supplementary Data 1: Characteristics of recovered MAGs.

Supplementary Data 2: Custom selected KEGG genes used to define metabolic pathways.

Supplementary Data 3: The metabolic profiles of all MAGs annotated by METABOLIC v4.0.
