# Supplementary material for: Metagenomic insights into microbial community structure and metabolism in alpine permafrost on the Tibetan Plateau
Source: Nat Commun. 2024 Jul 14;15:5920. doi: 10.1038/s41467-024-50276-2 (PMC11247091; doi:10.1038/s41467-024-50276-2)
Supplement: Supplementary file 7 — Reporting Summary [file 41467_2024_50276_MOESM7_ESM.pdf]

nature portfolio

Corresponding author(s): Yuanhe Yang

Last updated by author(s): Jun 17, 2024

Reporting Summary

Nature Portfolio wishes to improve the reproducibility of the work that we publish. This form provides structure for consistency and transparency in reporting. For further information on Nature Portfolio policies, see our Editorial Policies and the Editorial Policy Checklist.

Statistics

For all statistical analyses, confirm that the following items are present in the figure legend, table legend, main text, or Methods section.

n/a	Confirmed	
☐	☒	The exact sample size (*n*) for each experimental group/condition, given as a discrete number and unit of measurement
☐	☒	A statement on whether measurements were taken from distinct samples or whether the same sample was measured repeatedly
☐	☒	The statistical test(s) used AND whether they are one- or two-sided<br>*Only common tests should be described solely by name; describe more complex techniques in the Methods section.*
☒	☐	A description of all covariates tested
☐	☒	A description of any assumptions or corrections, such as tests of normality and adjustment for multiple comparisons
☐	☒	A full description of the statistical parameters including central tendency (e.g. means) or other basic estimates (e.g. regression coefficient) AND variation (e.g. standard deviation) or associated estimates of uncertainty (e.g. confidence intervals)
☐	☒	For null hypothesis testing, the test statistic (e.g. *F*, *t*, *r*) with confidence intervals, effect sizes, degrees of freedom and *P* value noted<br>*Give P values as exact values whenever suitable.*
☒	☐	For Bayesian analysis, information on the choice of priors and Markov chain Monte Carlo settings
☐	☒	For hierarchical and complex designs, identification of the appropriate level for tests and full reporting of outcomes
☐	☒	Estimates of effect sizes (e.g. Cohen's *d*, Pearson's *r*), indicating how they were calculated

Our web collection on statistics for biologists contains articles on many of the points above.

Software and code

Policy information about availability of computer code

Data collection	No software was used for data collection.
Data analysis	Spatial interpolation procedure was conducted within ArcMap 10.2. The amplicon and metagenomic data were analyzed using the following software: Vsearch v2.15.2, MUSCLE 5.1, FastTree 2.1.10, fastp v0.21.0, megahit v1.2.9, Prodigal v2.6.3, CD-HIT v.4.8.1, Salmon v1.5.1, eggnog-mapper v 2.1.3, metaWRAP v1.3.2, dRep v3.4.3, checkM v1.2.2 , GTDB-Tk 2.1.1 and METABOLIC v4.0. All statistical analyses were performed using R 4.0.3. The R packages used in this study are phyloseq, picante, ggpubr, Hmisc, vegan, iCAMP, edgeR. The R code used for the statistical analyses is available at https://github.com/kangluyao/Microbes_in_Tibetan_permafrost and https://doi.org/10.5281/zenodo.11648114.

For manuscripts utilizing custom algorithms or software that are central to the research but not yet described in published literature, software must be made available to editors and reviewers. We strongly encourage code deposition in a community repository (e.g. GitHub). See the Nature Portfolio guidelines for submitting code & software for further information.

Data

Policy information about availability of data

All manuscripts must include a data availability statement. This statement should provide the following information, where applicable:
- Accession codes, unique identifiers, or web links for publicly available datasets
- A description of any restrictions on data availability
- For clinical datasets or third party data, please ensure that the statement adheres to our policy

The 16S rRNA gene sequence data and the metagenomic sequence data have been deposited in the NCBI Sequence Read Archive (SRA) database under the

BioProject number PRJNA1037019. Data for the main results and supplementary files (Supplementary Data 1-3) of this study are publicly available from https://doi.org/10.5281/zenodo.11829921. Source data are provided with this paper.

Research involving human participants, their data, or biological material

Policy information about studies with human participants or human data. See also policy information about sex, gender (identity/presentation), and sexual orientation and race, ethnicity and racism.

Reporting on sex and gender	n/a
Reporting on race, ethnicity, or other socially relevant groupings	n/a
Population characteristics	n/a
Recruitment	n/a
Ethics oversight	n/a

Note that full information on the approval of the study protocol must also be provided in the manuscript.

Field-specific reporting

Please select the one below that is the best fit for your research. If you are not sure, read the appropriate sections before making your selection.

☐ Life sciences ☐ Behavioural & social sciences ☒ Ecological, evolutionary & environmental sciences

For a reference copy of the document with all sections, see nature.com/documents/nr-reporting-summary-flat.pdf

Ecological, evolutionary & environmental sciences study design

All studies must disclose on these points even when the disclosure is negative.

Study description	We set up 24 sites along an about 1,000 km permafrost transect and collected soil samples from 0-10 cm, 30-50 cm, and the uppermost 50 cm thick permafrost layer on the Tibetan alpine permafrost. After that, we employed both amplicon and metagenomic sequencing methods to examine the microbial community structure and functional profiles. Additionally, we retrieved climatic and vegetable factors, and determined the environmental variables including substrate properties and edaphic factors to explore the underlying drivers of microbial compositional variations.
Research sample	Soil samples were collected from 24 sites along an about 1,000 km permafrost transect on the Tibetan alpine permafrost. The reason we chose 24 sites is because they can represent the permafrost region across the plateau. Since two of these sites there were problems obtaining a sufficient DNA yield during DNA extraction and so only samples from 22 sites were processed in this study.
Sampling strategy	Soil samples were collected from 24 sites along an about 1,000 km permafrost transect on the Tibetan alpine permafrost. A 10 m × 10 m plot with five 1 m × 1 m quadrats along the diagonal line was established in each site. We utilized a borehole drilling apparatus to extract soil cores within each quadrat. Soil samples in the active layer were collected at the depth of 0-10 cm (SUR: surface) and 30-50 cm (SUB: subsurface). Simultaneously, we collected permafrost samples (PL: permafrost layer) from the uppermost 50 cm thick layer. Soil samples from each site and each layer were homogenized. Afterwards, the composite soils were then divided into two parts: one part was subjected to air-drying for subsequent soil physicochemical measurements, the other was preserved at -80℃ for subsequent DNA extraction.
Data collection	Field data including coordinate informations was collected during the field work by IBCAS Sampling Team using a pencil. Additionally, data about the climatic, anthropogenic and vegetable factors were retrieved from the public databases according to the coordinate informations. The environmental variables including substrate properties and edaphic factors were measured in lab. Data collection process involved the use of commercial software provided with the respective instruments.
Timing and spatial scale	We collected soil samples along an about 1,000 km permafrost transect across the Tibetan Plateau during July to August 2016, .
Data exclusions	Soil samples from two sites were discarded due to the insufficient DNA yield during DNA extraction. Therefore, only samples from 22 sites were ultimately processed in our study. We have clearly stated this point in our Methods section. No other data were excluded from the analysis.
Reproducibility	The study design, sampling method, lab measurements, and statistics are depicted in detail. All data and codes for reproducing the results are available publicly.
Randomization	In this study, twenty-two sites were randomly selected along an ~1,000 km permafrost transect on the Tibetan Plateau. In each site, five replicate soil samples for each soil layer (i.e. surface, subsurface, and permafrost layers) within the 10 m × 10 m plot were homogenized to represent the average condition at each site. Finally, twenty-two soil samples in each soil layers across the permafrost transect were used for our further analysis.
Blinding	The data collection and analysis were conducted using standardized protocols and automated methods, ensuring objective and

unbiased results. Therefore, blinding was not necessary as the study design inherently minimized the potential for observer bias or influence on the outcomes.

Did the study involve field work? ☒ Yes ☐ No

Field work, collection and transport

Field conditions	Soil samples were collected at 24 sites along an ~1,000 km permafrost transect on the plateau. The mean annual air temperature across all sites ranges from -4.5 to 1.8°C, and the mean annual precipitation varies across 245-504mm.
Location	In 2016, soil samples were collected at 24 sites with a longitude range of 91.86-99.50E and a latitude range of 34.02-38.58N along an ~1,000 km permafrost transect on the plateau (Fig. 1a). The elevation of these sites range from 3754-5141 m.
Access & import/export	Most of sites located in the three-river-source national park and Qinghai Hoh Xil National Natural Reserve. We obtained permission from these two national park administration to enter the area for sampling, and the soil samples were maintained in a frozen state and transported to the laboratory.
Disturbance	All sampling sites are located in remote and sparsely populated areas, most of which are above 4,000 meters and uninhabited. The values of human footprint among our sampling sites were lower than those in typical cities on the Tibetan Plateau, suggesting that most of sampling sites were subjected minor human disturbance (Supplementary Fig. 10).

Reporting for specific materials, systems and methods

We require information from authors about some types of materials, experimental systems and methods used in many studies. Here, indicate whether each material, system or method listed is relevant to your study. If you are not sure if a list item applies to your research, read the appropriate section before selecting a response.

Materials & experimental systems

n/a	Involved in the study	
☒	☐	Antibodies
☒	☐	Eukaryotic cell lines
☒	☐	Palaeontology and archaeology
☒	☐	Animals and other organisms
☒	☐	Clinical data
☒	☐	Dual use research of concern
☒	☐	Plants

Methods

n/a	Involved in the study	
☒	☐	ChIP-seq
☒	☐	Flow cytometry
☒	☐	MRI-based neuroimaging

Plants

Seed stocks	n/a
Novel plant genotypes	n/a
Authentication	n/a
